# Injury Epidemiology among Malaysian National Men's and Women's Football Teams: A Prospective Cohort Study during 2022-2023

**DOI:** 10.5704/MOJ.2603.015

**Published:** 2026-03

**Authors:** AH Ahmad-Shushami, MAI Rosidi, MS A-Hamid, KH Hussein, I Saudi

**Affiliations:** 1 Department of Sports Medicine, Universiti Malaya, Kuala Lumpur, Malaysia; 2 Department of Sports Medicine, National Sports Institute, Kuala Lumpur, Malaysia; 3 Department of Social and Preventive Medicine, Universiti Malaya, Kuala Lumpur, Malaysia

**Keywords:** football, national team, injury epidemiology

## Abstract

**Introduction:**

To determine the injury incidence, burden, and characteristics among Malaysian national football players.

**Materials and methods:**

Three Malaysian national men’s teams (U-19, U-23, and Senior) and the three Malaysian national women’s teams (U-18, U-20, and Senior) were followed prospectively from June 2022 to June 2023. All training and match injuries, together with exposures, were recorded by the team doctor and physiotherapist.

**Results:**

The men’s team recorded a total of 85 injuries during 5153 hours of exposure, equal to an incidence rate of 16.5 injuries per 1000 hours. Match injuries showed greater incidence (65.9 vs 7.8 injuries per 1000 hours) and burden (444.6 time-loss days per 1000 hours against 13.9 time-loss days per 1000 hours) as compared to training. Thigh injuries were the predominant injuries, followed by knee and ankle injuries. In the women’s team, 17 injuries were reported over 1698 hours of exposure, producing a total incidence rate of 10.0 injuries per 1000 hours. Higher injury frequency and burden were seen during match play (47.6 versus 4.1 injuries per 1000 hours and 1272.7 versus 6.81 time-loss days per 1000 hours, respectively). The ankle and quadriceps were the most frequently reported injuries, followed by the knee.

**Conclusion:**

The Malaysian national men’s team recorded a higher injury incidence compared to the women's, however no significant difference in injury burden noted between genders. The incidence of injuries during matches is greater than that during training, with thigh, ankle, and knee injuries being the most prevalent.

## INTRODUCTION

Football is a contact sport characterised by high-intensity physical demands. The game involves repeated changes of direction, acceleration, and deceleration movements among players, and the speed of the game is increasing in comparison to previous years^1,2^. It predisposed the players to a certain risk of injuries and may result in time lost from the training or matches^3^.

Based on the sports injury prevention model by van Mechelen, injury surveillance is the first crucial stage in an injury prevention programme^4^. This critical step improves understanding of the injury risk and pattern, allowing for the implementation of an appropriate injury prevention programme. In 2006, an international consensus statement was released, which outlined injury surveillance standards to ensure uniformity, methodical, and thorough reporting^5^.

Over the years, numerous studies of injury epidemiology have been conducted all over the world. The incidence of injuries encountered by football players differs between countries due to variances in playing styles and climate^6,7^. A comprehensive research project that studies over 18 years of data from European football clubs revealed an overall decline in the incidence of injuries^8^. This suggests that injury prevention strategies are effective, and that continuous effort should be made to prevent injuries. Nevertheless, these findings are limited to professional football players in Europe. A prospective study among professional football players in Asia showed comparable injury rate to the European counterparts, however, the incidence of ACL and hamstring injuries were reported to be higher^9^.

While most studies focus on injury in professional football players, limited information is available on injury incidence among national football players, particularly in Asia^10,11^. Moreover, comparing injuries across different playing levels is essential as the intensity and demands of international competition often differ significantly from those in national or domestic leagues, potentially leading to different patterns of injuries^12-14^. An analysis of the Australian women’s team revealed that, compared to players in national leagues, the total distances covered and high-speed running distances were significantly greater in international football^14^. The transition from club level to the national football team led to an anticipated rise in the acute-to-chronic workload ratio, largely due to the rapid increase in training sessions over a short period^13^. The study also found that injured players experienced higher internal and external loads compared to non-injured players during the transition from club to national camp in preparation for the international match^13^.

Despite the significant differences between domestic competitions and international football, research on injuries among national football players, particularly in Asia, is still scarce^10,11^.

This study aims to determine the injury incidence, burden, and characteristics among the Malaysian national men's and women's football players.

## MATERIALS AND METHODS

A prospective cohort study design was used to determine the injury incidence, burden, and characteristics among the Malaysian national football players.

The study involved football players from six Malaysian national football teams, including the men's under-19 (U-19), under-23 (U-23), and senior teams, as well as the women's under-18 (U-18), under-20 (U-20), and senior teams. Data were collected prospectively over the course of a year, from June 2022 to June 2023. This included a detailed and systematic recording of injuries, match participation, and training exposure among the athletes representing these national teams.

The Malaysian national team medical staff (team physicians and physiotherapists) of the respective teams attended a half-day workshop on injury recording using the standardised injury report form as recommended by the international consensus^5,15^. Football players’ baseline and sociodemographic information including age, height, weight, and playing position were collected using a clinical research form.

Information on match and training time exposures was obtained from the respective coaching teams. Match exposure was defined as participation in a competitive match with another team. The calculation involved multiplying the number of players^11^ by total match time (in minutes) and subsequently dividing by 60 to express the results into hours. Training exposure included both team training and supervised (coaching staff) individual physical activities sessions. The training exposure was determined by multiplying the number of players by the training duration (in minutes) and dividing by 605.

The injury definition utilised in this study was based on a widely accepted consensus, encompassing both medical attention injuries, which referred to all injuries attended by the medical team, and time-loss injuries that prevent the player from participating in future training sessions or matches^5,15^. Injury severity was categorised based on time away from participation as mild (1 to 3 days of absence), minor (4 to 7 days), moderate (8 to 28 days), and major (more than 28 days)^5^.

Before participating in the study, all players were required to sign an informed consent form. For players under the age of 18, written consent was also obtained from their parents or guardians. Ethical approval for the study was granted by the Medical Research and Ethics Committee (MREC) of the University of Malaya Medical Centre.

Continuous data were described as means ± SD or median ± IQR based on data distribution. To ensure a thorough comparison and maintain methodological rigor, the analysis was stratified by sex. This approach was taken to account for potential differences in injury patterns between male and female players.

Injury incidence and burden were calculated using standardised formulas to ensure consistency and comparability of the results. The injury incidence rate was measured as the number of injuries per 1000 athlete-exposure hours, using the following formula^5^: Injury Incidence = (total number of injuries×1000) ⁄ total exposure hours while injury burden was defined as^16^: Injury Burden = (total number of days absent×1000) ⁄ total exposure hours

Based on the data distribution the Mann-Whitney U test was utilised to compare injury metrics (injury incidence and injury burden) between sex and team categories. Statistical significance was set at a p-value of <0.05. The 95% confidence intervals for injury burden were calculated using bootstrapping methods. This process involved repeatedly resampling the data with replacement to generate multiple simulated samples, which provided robust interval estimates. All analyses were conducted using SPSS (IBM SPSS statistics version 25).

## RESULTS

A total of 448 football players from six national Malaysian football teams were recruited and monitored prospectively for a period of 12 months from June 2022 until June 2023. During this period, a total of 102 injuries were documented. The distribution of players and their engagement in training and matches varied across teams, as detailed in Table I, which also encapsulates the football players demographic and anthropometric characteristics.

**Table I:** Malaysian national footballers characteristics and playing exposures.

**National Teams**	**Men**			**Women**		
	**Senior**	**U-23**	**U-19**	**Senior**	**U-20**	**U-18**
Age (years)	26.8 ± 4.1	20.9 ± 1.2	18.5 ± 0.7	24.3 ± 5.0	17.1 ± 1.3	17.3 ± 0.5
Height (cm)	177.3 ± 7.9	176.2 ± 6.8	173.6 ± 6.1	157.5 ± 6.1	157.0 ± 5.4	158.1 ± 4.1
Weight (kg)	70.9 ± 9.0	69.4 ± 7.2	66.4 ± 6.0	53.5 ± 5.7	51.0 ± 5.4	52.2 ± 4.4
BMI	22.5 ± 1.4	22.3 ± 1.6	22 ± 1.6	21.6 ± 2.1	20.6 ± 1.3	20.8 ± 1.4
Number of Players	110	132	96	55	25	30
Number of Training	51 (75%)	44 (73%)	29 (69%)	13 (62%)	16 (84%)	12 (80%)
Number of Matches	17 (25%)	16 (27%)	13 (31%)	8 (38%)	3 (16%)	3 (20%)

Notes - Values are mean ± standard deviation (SD) unless otherwise stated. Number in bracket is percentage of total number of exposure activity. BMI: body mass index, U-23: under 23, U-20: under 20, U-19: under 19, U-18: under 18

Over the study period, a total of 6851 hours of exposure, comprising 1004 hours of matches and 5847 hours of training, were recorded by six national Malaysian football teams. During this period, 60 matches (tournament and friendly) and 166 training sessions were included in this study (Table I).

The men’s teams reported a total of 85 injuries, translating to a total injury incidence rate of 16.5 per 1000 hours. Majority (n=51) of the injuries involved time loss, equating to 10.7 injuries per 1000 hours. A significantly higher incidence rate (p<0.05) was recorded during match play with 65.9 injuries per 1000 hours compared to 7.8 injuries per 1000 hours in training sessions. Conversely, the women’s teams encountered 17 injuries throughout the study, yielding in an overall injury incidence rate of 10.0 injuries per 1000 athlete exposure hours. Of these, 9 were time-loss injuries, equating to 5.3 injuries per 1000 hours. Similar to the men’s teams, the injury incidence during matches (47.6 injuries per 1000 hours) was significantly higher (p<0.05) than during training (4.1 injuries per 1000 hours).

A clear difference in injury burden was noted between match play and training sessions. For the men’s teams, the overall injury burden was 66.8 time-loss days per 1000 hours of sports activity. During matches, the injury burden was significantly higher at 444.6 time-loss days per 1000 hours, compared to 13.9 days per 1000 hours during training (p<0.001).

Similarly, the women’s teams experienced an overall injury burden of 179 time-loss days per 1000 hours. The injury burden during matches was notably higher at 1272.7 time-loss days per 1000 hours, significantly surpassing injury burden during training at 6.81 time-loss days per 1000 hours (p<0.001). Table II displays the injury incidence and burden among Malaysian national football players.

**Table II:** Injury incidence and burden among Malaysian national football teams.

**National Team**		**Men**			**Women**		
		**Senior**	**U-23**	**U-19**	**Senior**	**U-20**	**U-18**
Number of Injury, n (%)	Total	32	35	18	7	5	5
	Time-loss injury	21	27	7	5	2	2
	Match	19 (59%)	21 (60%)	11 (61%)	5 (71%)	3 (60%)	3 (60%)
	Training	13 (41%)	14 (40%)	7 (39%)	2 (29%)	2 (40%)	2 (40%)
Exposure (hours)	Total	2034.7	1859.3	1260.0	617.0	582.0	499.0
	Match	284.2	269.5	220.0	132.0	49.5	49.5
	Training	1750.5	1589.8	1040.0	485.0	532.5	449.5
Time loss injury (days)	Total	133	150	61	294	2	8
	Match	80	80	54	287	2	5
	Training	53	70	7	7	0	3
Injury Incidence (95% CI)	Total	15.7	18.8	14.3	8.1	8.6	10.0
	Time-loss injury	10.32	14.52	5.56	8.10	3.44	4.01
	Match	66.9	78.0	50.0	37.9	60.6	60.6
	Training	7.4	8.8	6.7	4.1	3.8	4.5
Injury Burden (95% CI)	Total	65.4	80.7	48.4	476.5	3.4	16.0
	Match	281.5	296.9	245.5	2174.2	40.4	101.0
	Training	30.3	44.0	6.73	14.4	0	6.7

Notes - Number in bracket is percentage of total number of injuries. U-23: under 23, U-20: under 20, U-19: under 19, U-18: under 18.

The greater number of players on the men's teams compared to the women's teams is attributed to the inclusion of extended squads and player rotations during international competitions. This approach ensures comprehensive injury surveillance and player availability for multiple matches and training sessions. All players, including those on extended squads, were involved in match minutes, reflecting the competitive and dynamic nature of national team selections. Further analysis was conducted to explore sex disparities in injury patterns. The results showed significantly higher injury incidence among male compared to female players (p<0.05). However, the analysis of injury burden did not demonstrate a significant difference between sex (W=595, p=0.2347). This suggests that the overall impact of injuries, in terms of time lost from sport, is comparable for both male and female players.

The most frequently injured regions among men were the thigh (4.3 injuries per 1000 hours), followed by the knee (2.7 injuries per 1000 hours), and the ankle (2.5 injuries per 1000 hours). Hamstring muscle strains were the most frequent at 16.5%, followed by ankle ligament sprains (15.3%), and knee ligament sprains (10.5%) (Table III).

**Table III:** Data on the injury pattern and burden men’s national team.

**Body region / Injury type and**	**Number of injuries n (%)**	**Median Time loss Days (95% CI)**	**Injury Incidence (95%CI)**	**Injury Burden (95%CI)**
Head/face	4 (4.7)	3.5	0.78	0.68
Facial Laceration Wound	2(2.4)	0	0.39	0.00
Concussion	2 (2.4)	7	0.39	1.36
Neck/ cervical spine	2 (2.4)	2	0.39	0.39
Muscle strain	2 (2.4)	2	0.39	0.39
Upper arm	1 (1.2)	0	0.19	0.00
Contusion	1(1.2)	0	0.19	0.00
Elbow	1 (1.2)	0	0.19	0.00
Abrasion wound	1 (1.2)	0	0.19	0.00
Wrist and hand	4 (4.7)	16.5	0.78	3.20
Sprain	2 (2.4)	2.5	0.39	0.49
Fracture	2 (2.4)	30	0.39	5.82
Chest and Thoracic Spine	2 (2.4)	1	0.39	0.19
Contusion	2 (2.4)	1	0.39	0.19
Lumbosacral	2 (2.4)	2	0.39	0.39
Contusion	1 (1.2)	2	0.19	0.39
Muscle strain	1 (1.2)	2	0.19	0.39
Abdomen	3 (3.5)	0	0.58	0.00
Muscle strain	3 (3.5)	0	0.58	0.00
Hip	1 (1.2)	3	0.19	0.58
Contusion	1 (1.2)	3	0.19	0.58
Groin	5 (5.9)	3	0.97	0.58
Muscle strain	5 (5.9)	3	0.97	0.58
Thigh	22 (25.9)	3	4.27	0.58
Hamstring muscle strain	14 (16.5)	4.5	2.72	0.87
Hamstring muscle contusion	1 (1.2)	1	0.19	0.19
Quadriceps muscle strain	4 (4.7)	3	0.78	0.58
Quadriceps muscle contusion	4 (4.7)	0	0.78	0.00
Knee	14 (16.5)	3	2.72	0.58
Ligamentous sprain	9 (10.6)	5.5	1.75	1.07
Contusion	5 (5.9)	0	0.97	0.00
Lower leg	6 (7.1)	1	1.16	0.19
Contusion	4 (4.7)	1	0.78	0.19
Muscle strain	2 (2.4)	1.5	0.39	0.29
Ankle	13 (15.3)	3	2.52	0.58
Ligamentous sprain	13 (15.3)	3	2.52	0.58
Foot	3 (3.5)	2	0.58	0.39
Contusion	3 (3.5)	2	0.58	0.39

**Fig. 1:**
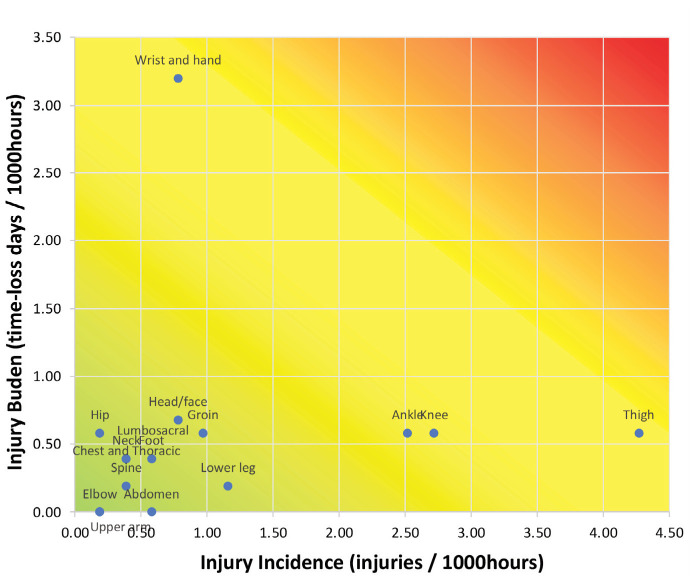
Risk matrix of men’s injury pattern.

Fig. 1 illustrates the injury risk matrix, which depicts the relationship between injury incidence and injury burden by body region. Darker shades represent a higher risk of injury. Among men, thigh injuries showed the highest risk matrix, followed by knee and ankle injuries, indicating that the injury has a high incidence rate as well as a high burden.

**Fig. 2:**
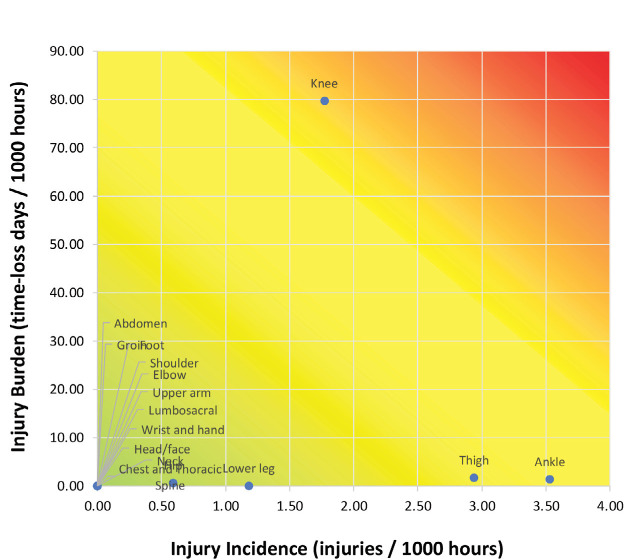
Risk matrix of women’s injury pattern.

The ankle was the most frequent region of injury among women (3.5 per 1000 hours), followed by the thigh (2.9 per 1000 hours) and the knee (1.8 per 1000 hours). To be more specific, ankle ligament sprain was the most frequent injury diagnosis (35.3%), followed by knee ligament sprain (17.6%), and hamstring strain (17.6%) (Table IV). The risk matrix for women’s football players is shown in Fig. 2. In contrast to men, knee injuries among women were associated with a higher burden, despite a lower incidence rate.

**Table IV:** Data on the injury pattern and burden of women’s national team.

**Body region / Injury type and diagnosis**	**Number of injuries (n)** **(%)**	**Median time loss days** **(95% CI)**	**Injury incidence** **(95%CI)**	**Injury burden** **(95%CI)**
Hip				
Contusion	1 (5.9)	10.59	0.59	
Thigh	5 (29.4)	32.94	1.77	
Hamstring muscle strain	3 (17.6)	31.77	1.77	
Quadriceps muscle contusion	2 (11.8)	01.18	0.00	
Knee				
Ligamentous sprain	3 (17.6)	135.5	1.77	79.80
Lower leg				
Contusion	2 (11.8)	01.18	0.00	
Ankle				
Ligamentous sprain	6 (35.3)	2.5	3.53	1.47

## DISCUSSION

This study revealed that men and women national football players experienced a greater injury rate during matches than training. This result aligned with the findings of the previous research, which indicated a higher incidence of injuries sustained during matches^8,17,18^. The injury incidence was greater in men’s teams than in women’s teams. However, our analysis showed no significant difference in injury burden between men and women football players. Notably, the women's senior squad had the highest injury burden.

This study reported a higher incidence of injury among men (16.5 injuries per 1000 hours) compared to a similar study conducted on a national team from another Asian country which used similar injury definition including regardless of time loss and found 7.8 injuries per 1000 hours^10^. The injury incidence in this study was also greater than that observed in a study of injury epidemiology in 13 men’s international football teams conducted in Europe, which reported 9.8 injuries per 1000 hours^19^.

The lower injury incidence reported in prior research may be attributed to the fact that only injuries resulting in time loss were documented^9^. Moreover, greater injury rate in this study could be attributed to the heightened competitiveness and intensity of play within a condensed timeframe, where coaches struggle with player rotation during tournaments and international matches^20-23^. These findings align with previous research suggesting that national team players may be at greater risk of injury due to differences in team dynamics and shorter training periods^24^.

Compared to a similar systematic review and meta-analysis, our study reported a greater 65.9 injuries per 1000 hours vs 32.3 injuries per 1000 hours), and higher injury rate during training (7.76 injuries per 1000 hours vs 3.8 injuries per 1000 hours)^19^. This difference could be attributed to the excessive number of matches occurring between international competitions and domestic leagues, particularly since some of the international tournaments are not officially scheduled by FIFA. Prior research has identified match congestion as a risk factor that contributes to an elevated risk of injury due to inadequate recuperation between matches^20-22^.

This study found that the overall injury burden for the national men’s team was 66.8 time-loss days per 1000 hours, with significantly higher burden during matches (444.6 time-loss days per 1000h) compared to training (13.9 time-loss days per 1000h). These results are comparable to a study in England, which documented a match injury burden of 454 time-loss days per 1000 hours and a training injury burden with 51.0 time-loss days per 1000h)^16^. Injury burden, which reflects the amount of time a player is unavailable due to an injury, offers a valuable perspective in injury documentation and helps understand how injuries impact the team.

Lower limb injuries were common among Malaysian men’s international football teams with the thigh, knee, and ankle being the most frequently affected areas. Thigh injuries especially hamstring muscle strains, were the most prevalent and resulted in a significant injury burden. These findings are consistent with previous research involving professional football players^10,11,23^. The high occurrence of lower limb injuries among football players can be attributed to the nature of the sport, which primarily relies on lower extremity movements, particularly involving the feet. Football players frequently engage in high-intensity drills including sprinting, turning, leaping, landing, and tackling which can cause lower limb fatigue. Repeated fatigue is thought to increase the likelihood of these can lead to lower limb injuries^24^.

Injury surveillance studies in women's football have shown promising results, with a growing focus on injury prevention in this once male-dominated sport^16,17,25^. Our findings revealed an overall injury incidence of 10.0 injuries per 1000 hours in women’s football, with 47.6 injuries per 1000 hours occurring during matches. The match injury rate in international women’s football in this study is lower compared to previous meta-analyses (67.4 per 1000h)^24^. The lower injury rate may be due to the reduced level of game intensity in this region.

The match injury burden for the national women’s teams was significantly higher at 1272.7 time-loss days per 1000 hours compared to the training injury burden of 6.81 time-loss days per 1000 hours. This match injury burden was substantially greater than that reported in a previous study on injury burden in women’s international football (506.7+350.2 time-loss days per 1000h)^16^. Although the injury incidence rate in this study was lower than in the previous study^24^, the higher injury burden was due to an anterior cruciate ligament injury that required surgery. Therefore, it is crucial to assess both injury incidence and burden to fully understand the overall impact of injuries.

Analysis of injury patterns among the Malaysian national women's football teams revealed that ankle injuries occur most frequent, occurring at a rate of 4.0 injuries per 1000 hours. These injuries also carried a significant burden, with 5.0 time-loss days per 1000 hours, compared to other body parts. This observation is consistent with findings from other studies on elite women's football, which also identified the ankle as the most frequently injury area^17,25^.

The higher match injury incidence in the women's U-20 and U-18 teams compared to the women's senior team is noteworthy. Several factors may explain this difference, including the lower number of games played by the junior teams. In contrast, a previous study found no significant difference in demonstrated injury incidence and burden between senior and youth teams but noted that these rates can fluctuate from season to season^16^. Other contributing factors include differences in playing style, level of competition and the number of matches^16^.

According to the injury risk matrix in women's teams, lower extremity injuries were the most frequent. The thigh, knee, and ankle were the most frequent injured sites, consistent with findings from previous studies in women's football^17,18,26^. In this study, the knee injuries were identified as high-risk given their significant burden and high incidence rate. Prior research has shown that the incidence of ACL injuries is higher in female compared to male athletes^27^. Both modifiable and non-modifiable risk factors contribute to these sex differences in ACL injury risk, including anatomical differences, hormonal influences, and landing biomechanics^27^. Therefore, sports medicine clinicians should consider these sex-specific risks when developing tailored injury prevention strategies.

Compared to men, women reported a higher injury burden despite experiencing fewer injuries overall. Men had a higher incidence rate if injuries, while women faced a greater burden when injured. This indicates that sex may influence injury occurrence. These results are in line with previous systematic reviews that found a higher overall incidence among men compared to women^28^. However, the degree of difference is influenced by the type of sports.

Although women had a injury burden (179 time-loss days per 1000h) compared to men (66.8 time-loss days per 1000h), this difference was not statistically significant. Previous studies have shown a similar comparison in injury burden (506.7 time-loss days per 1000h for men and 454.0 time-loss days per 1000h) for women. Due to known anthropometric and physiological variations between sexes, this variation in injury burden was not explored further^16^.

It should be highlighted that in this study, medical support and availability were standardised across all teams to minimise difference in injury reporting. However, evidence suggests that female athletes might still face disparities in medical support and under-reporting. Female athletes often receive fewer resources, and less support compared to their male counterparts, including reduced access to specialised medical professionals trained to address their specific health needs^27,28^. Additionally, cultural factors and less media attention might contribute to under-reporting of injuries in women's teams^27,28^.

This study offers important contributions to the field of sports injury epidemiology; however, it is not without limitations. The data collection relied on reports from the coaching and medical teams, which may introduce bias and affect accuracy. Additionally, the rotational nature of national team call-ups might affect the number of players selected for the study. There is also a possibility of underreporting, particularly for the women's team, as the injury data for the men's team seems significantly larger. Future studies could address this issue by utilising GPS systems for gather real-time, objective data on player activities. Although the study's met minimum duration standards, the observation period was relatively short. Extending the study across multiple seasons would allow for a comprehensive analysis of injury patterns. Additionally, the frequent player rotations within the national team also suggests that a longitudinal approach, tracking individual players might provide a clearer understanding of injury dynamics.

To address these limitations, future research should focus on exploring the underlying causes of the injury patterns identified in this study. This could include an in-depth analysis of training loads, biomechanical assessments, and the impact of recovery protocols on injury incidence and severity. Understanding these factors is essential for developing targeted interventions that could significantly reduce the injury risk in football, ultimately improving both player welfare and team performance. We would like to recommend that stakeholders in Malaysian football actively engage in more research and development programs in the future to raise the level of football medicine practice in this region.

## CONCLUSION

The Malaysian national men’s team recorded a higher injury incidence compared to women, but the difference in injury burden between the sexes was not statistically significant. Injuries sustained during matches were more frequent and had a greater burden compared to those occurring during training. Lower limb injuries were the most common, with the thigh injuries being the most frequent among men and ankle injuries the most frequent among women. This study provides valuable insights into injuries among Malaysian national football players at the international level, offering essential guidance for practitioners to develop injury prevention strategies based on risk mitigation.

## CONFLICT OF INTEREST

The authors declare no potential conflict of interest.

## ACKNOWLEDGEMENT

We extend our deepest gratitude to the National Sports Institute for their support. We are also immensely thankful to the Football Association Malaysia (FAM) for supporting our research project. A special thanks goes to the FAM medical team from for their assistance with data management.
